# Integration-Free iPS Cells Engineered Using Human Artificial Chromosome Vectors

**DOI:** 10.1371/journal.pone.0025961

**Published:** 2011-10-05

**Authors:** Masaharu Hiratsuka, Narumi Uno, Kana Ueda, Hajime Kurosaki, Natsuko Imaoka, Kanako Kazuki, Etsuya Ueno, Yutaro Akakura, Motonobu Katoh, Mitsuhiko Osaki, Yasuhiro Kazuki, Masato Nakagawa, Shinya Yamanaka, Mitsuo Oshimura

**Affiliations:** 1 Division of Molecular and Cell Genetics, Department of Molecular and Cellular Biology, School of Life Sciences, Faculty of Medicine, Tottori University, Yonago, Japan; 2 Department of Biomedical Science, Institute of Regenerative Medicine and Biofunction, Graduate School of Medical Science, Tottori University, Yonago, Japan; 3 Chromosome Engineering Research Center, Tottori University, Yonago, Japan; 4 Division of Human Genome Science, Department of Molecular and Cellular Biology, School of Life Sciences, Faculty of Medicine, Tottori University, Yonago, Japan; 5 Center for iPS Cell Research and Application, Kyoto University, Kyoto, Japan; 6 Japan Science and Technology Agency, CREST, Tokyo, Japan; The University of Manchester, United Kingdom

## Abstract

Human artificial chromosomes (HACs) have unique characteristics as gene-delivery vectors, including episomal transmission and transfer of multiple, large transgenes. Here, we demonstrate the advantages of HAC vectors for reprogramming mouse embryonic fibroblasts (MEFs) into induced pluripotent stem (iPS) cells. Two HAC vectors (iHAC1 and iHAC2) were constructed. Both carried four reprogramming factors, and iHAC2 also encoded a p53-knockdown cassette. iHAC1 partially reprogrammed MEFs, and iHAC2 efficiently reprogrammed MEFs. Global gene expression patterns showed that the iHACs, unlike other vectors, generated relatively uniform iPS cells. Under non-selecting conditions, we established iHAC-free iPS cells by isolating cells that spontaneously lost iHAC2. Analyses of pluripotent markers, teratomas and chimeras confirmed that these iHAC-free iPS cells were pluripotent. Moreover, iHAC-free iPS cells with a re-introduced HAC encoding *Herpes Simplex virus* thymidine kinase were eliminated by ganciclovir treatment, indicating that the HAC safeguard system functioned in iPS cells. Thus, the HAC vector could generate uniform, integration-free iPS cells with a built-in safeguard system.

## Introduction

Reprogramming somatic cells to become induced pluripotent stem (iPS) cells is important in making regenerative medicine a reality [1]-[3]. The best iPS cells for therapeutic applications are derived from cells harvested from individual patients and the reprogramming should not involve permanent genetic changes because strategies involving insertional modifications of the genome increase the risk of insertional mutagenesis [4] and perturbation of differentiation potential [5]. To avoid permanent, detrimental modification of the host genome while reprogramming somatic cells, several vectors and protocols that exclude permanent transgene integration into the host genome have been developed: the piggyBac transposon [6]-[8], adenovirus vectors [9], Sendai virus vectors [10], EB-derived episomal vectors [11] and iterant administration of non-replicative materials (i.e. plasmid [12], minicircle DNA [13], protein [14], and synthetic modified mRNA [15]). However, these vectors and methods should be scrutinized with regard to quality of individual iPS cells, reprogramming efficiency and genome integrity. In addition, iPS cells should have a safeguard system because iPS cells with teratoma-forming potential can persist even after differentiation, leading to unexpected and undesired events [16].

With respect to the generation of iPS cells, human artificial chromosomes (HACs) have two important and unique characteristics as gene-delivery vectors; effectively unlimited carrying capacity for transgenic material and autonomous maintenance through cell division that is independent of host chromosomes. We have created several HAC vectors from human chromosome 21 using a top down method [17], [18] and have demonstrated that full-length genomic loci, such as DMD [19], HPRT [20] and p53 [20] could be cloned into a defined HAC cloning site. We have also shown that these loci are efficiently transcribed. Moreover, expression in human cells of cDNAs introduced into HACs was more stable and sustained and less subject to position effects [21] than expression of cDNAs from conventional plasmids and viral vectors. In addition, our HAC vectors encode EGFP [18]; therefore, because HACs are lost spontaneously at a low frequency [22] we can isolate HAC-free cells from reprogrammed iPS populations by identifying EGFP-negative cells.

Here, we have taken advantage of these features of HAC vectors to generate vector-free and transgene-free iPS cells. Recent attempts to generate iPS cells using polycistronic vectors to express multiple proteins demonstrated that a significant portion of the iPS clones carried more than two copies of the polycistronic vector [6], [8], [23], [24], suggesting that multiple copies of the polycistronic transgenes were needed to generate iPS cells. Thus, we devised a reprogramming cassette with four defined reprogramming factors and introduced multiple copies of the cassette into the cloning site of a HAC vector. We constructed a closely related cassette by adding a p53 short hairpin RNA (shRNA) expression construct to the four-factor cassette because suppression of the p53 pathway leads to more efficient reprogramming [25]-[29]. Moreover, our HAC vector encodes *Herpes Simplex virus* thymidine kinase (*HSV*-TK), and we confirmed that iPS cells and/or their differentiated derivatives carrying our HAC can be killed by ganciclovir (GCV), providing a safeguard system if unexpected events (e.g., tumor formation) occur.

## Methods

### Ethics Statement

All animal experiments were approved by the Institutional Animal Care and Use Committee of Tottori University (the permit number: 08-Y-69).

### Plasmid construction

We constructed individual expression cassettes for each reprogramming factor in pBSII (Stratagene) and combined all cassettes into a pPAC4 backbone as follows. The pBSII multiple cloning site was replaced with either KpnI-XhoI-AscI-BsiWI-NheI-ClaI-SalI-PstI-AvrII-PmeI-FseI-XbaI-SpeI-SacI or KpnI-XhoI-AscI-BsiWI-NheI-ClaI-SalI-MluI-SphI-SnaBI-NotI-SacII-BamHI-AvrII-PmeI-FseI-XbaI-SpeI-SacI, resulting in the vectors pB3 and pB4, respectively. Two different 1.2 kb fragments of the chicken HS4 insulator were excised by either SacI or XbaI digestion of pCJ5-4 (a gift from Dr. G. Felsenfeld, National Institutes of Health, Bethesda, MD, USA), blunted by KOD polymerase (Toyobo), and cloned into the SmaI site or the blunted HindIII site of pBSII, respectively. The resulting vector, harboring 2 copies of HS4, was called pBSI-I. A ClaI-BamHI fragment of pBSI-I was cloned into (1) a blunted ClaI site of pB3, (2) a blunted ClaI site of pB4, or (3) blunted ClaI and PmeI sites of pB4, resulting in (1) pinsB3, (2) pinsB4 and (3) pB4ins2, all of which retained the BamHI site immediately downstream of the HS4 dimer. All subcloned HS4 insulators had the same orientation.

Mouse Klf-4, c-Myc, Sox2 and Oct4 were PCR-amplified and individually cloned into the EcoRI site of pCAGGS (a gift from Dr. M. Okabe, Osaka University, Japan), resulting in pCX-Klf4, pCX-c-Myc, pCX-Sox2 and pCX-Oct3/4, respectively. SalI-BamHI fragments of pCX-Klf4, pCX-c-Myc and pCX-Sox2 were blunted and cloned into blunted BamHI sites of pinsB4, resulting in pB4K, pB4M and pB4S, respectively; an SspI-BamHI fragment of pCX-Oct3/4 was cloned into a SnaBI site of pB4ins2, resulting in pB4O. To combine four factors in a single vector, AscI-AvrII fragments from pB4K and pB4S were inserted into the AscI-NheI sites of pB4M and pB4O, resulting in pB4KM and pB4SO, respectively. Finally, an AscI-AvrII fragment of pB4KM and an NheI-FseI fragment of pB4SO were ligated into the AscI and FseI sites of pPH3-9, which was generated by modifying pPAC4; specifically we exchanged the region between the pUC link and the CMV promoter with HPRT ex3-ex9 and added an FseI site immediately downstream of HPRT ex9. The resulting vector was designated pPAC-KMSO. This KMSO reprogramming cassette was duplicated by the same strategy, resulting in pPAC-2CAG-KMSO. A fragment of the duplicated pB4O was cloned into the AscI-NheI site of pPAC-2CAG-KMSO, resulting in pPAC-2CAG-O2.

A mouse p53-knockdown construct was generated by annealing two complementary synthetic oligonucleotides with the target sequence GTACATGTGTAATAGCTCC and cloning the product into the BglII-XbaI sites of pENTR4-H1 (a gift from Dr. H. Miyoshi, RIKEN, Japan), resulting in pENTR4-H1-mp53sh. A SalI-XbaI fragment of pENTR4-H1-mp53sh was inserted into the SalI-AvrII site of pinsB3, resulting in pinsB3mp53sh. Finally, an AscI-SpeI fragment of pinsB3mp53sh was inserted into the AscI-NheI site of pPAC-2CAG-O2, resulting in pPAC-2CAG-O2mp53sh.

### Cell culture

Hprt-deficient Chinese hamster ovary cells (JCRB0218, JCRB Cell Bank, Japan) each bearing a HAC vector, (CHO(21HAC2), CHO/iHAC1/E15 and CHO/iHAC2/mp25) were maintained at 37°C in Ham’s F-12 nutrient mixture (Invitrogen) supplemented with 10% fetal bovine serum (FBS) and 8 µ/ml Blasticidin S (Funakoshi). Mouse embryonic fibroblasts (MEFs), isolated from 13.5 day post-coitum (d.p.c.) wild-type embryos (C57BL/6-J), were grown in Dulbecco’s modified Eagle’s medium (DMEM) (Sigma) plus 10% FBS. The mouse ES cell lines, TT2 (a gift from Dr. S. Aizawa, RIKEN, Japan) [30] and B6ES (DAINIPPON SUMITOMO PHARMA, Osaka, Japan), and the microcell hybrid clones, were maintained on mitomycin C-treated Jcl:ICR (CLEA Japan) MEF feeder layers in ES medium [DMEM with 18% FBS (Hyclone), 1 mM sodium pyruvate, 0.1 mM non-essential amino acids, 2 mM L-glutamine (Invitrogen), 0.1 mM 2-mercaptoethanol (Sigma), and 1000 U/ml leukemia inhibitory factor (LIF) (Millipore)].

### Construction of iHAC vectors

The reprogramming cassettes were introduced into 21HAC2 vectors using the Cre-loxP system. Suspensions of CHO(21HAC2) cells (5 × 10^6^ cells in PBS) were mixed with 10 µg of the Cre expression plasmid (pBS185) and 20 µg of pPAC-2CAG-KMSO. Electroporation was performed at 450 V and 500 µF using a GenePulser Xcell (Bio-Rad). pBS185 (1 µg) and pPAC-2CAG-O2mp53sh (2 µg) were transfected into semi-confluent CHO(21HAC2) cells in a 60 mm dish using Lipofectamine2000 (Invitrogen) according to the manufacturer’s instructions. These cells were treated with HAT (Sigma) and 8 µg/ml Blasticidin S two days after transfection. After 2-3 weeks, drug-resistant colonies with a functional HPRT allele were identified by genomic PCR and isolated.

### Microcell-mediated chromosome transfer (MMCT) and reprogramming of MEFs using iHAC vectors

MMCT was carried out as described previously [31]. CHO/iHAC1/E15 and CHO/iHAC2/mp25 were used as microcell donor cells. In brief, microcells were separated from donor cells by centrifugation and fused with MEFs in 45% polyethyleneglycol 1500 (Roche) and 10% dimethylsulfoxide (Sigma). The next day, fused cells were re-plated onto feeder layers. On day 2 and day 5, a 25 µM miR-294 and miR-295 cocktail (Dharmacon) was transfected using DharmaFECT 1 (Dharmacon) according to the manufacturer’s instructions. Culture medium was replaced with ES medium on day 3, and individual ES-like colonies were isolated from day 12 onwards.

### FISH analysis

FISH analyses were performed on either fixed metaphase or interphase spreads of each cell hybrid using digoxigenin-labeled (Roche) human COT-1 DNA (Invitrogen) and biotin-labeled pPAC-KMSO, essentially as described previously [31]. Images were captured using the NIS-Elements system (Nikon). We counted 20 metaphases and 100 interphases for each analysis.

### M-FISH analysis

Procedures for the denaturation of metaphase chromosomes and mFISH probes (MetaSystems), hybridization, post-hybridization washes and fluorescent staining were performed in accordance with the manufacturer's instructions. Metaphase images were captured digitally with a cooled CCD camera equipped with an ISIS mFISH software program (MetaSystems), processed and stored for subsequent analysis.

### RT-PCR analysis

Total RNA was extracted with TriZol (Invitrogen) and cDNA was synthesized using an oligo(dT) primer and ReverTra Ace (Toyobo). Quantitative RT-PCR was performed using the Power SYBR Green PCR Master Mix (Applied Biosystems) on an ABI7900HT (Applied Biosystems). Primer sequences are listed in Table S1.

### Immunohistochemistry

Cells were fixed with 4% paraformaldehyde in PBS at 4°C overnight and permeabilized and blocked in 0.1% Triton X-100, 5% normal goat serum (Millipore) and 5% skim milk in PBS for 15 min at room temperature. Primary antibodies used were rabbit anti-Nanog (1∶100, AB5731, Millipore), mouse monoclonal anti-Oct4 (1∶100, sc-5279, Santa Cruz), rabbit anti-α-fetoprotein (undiluted, N1501, Dako), mouse monoclonal anti-α-smooth muscle actin (undiluted, N1584, Dako) and rabbit anti-βIII-tubulin (1∶2000, PRB-435P, Covance). Secondary antibodies used were Alexa 594-conjugated goat anti-rabbit IgG (1∶1000, Invitrogen) and Alexa 594-conjugated goat anti-mouse IgG (1∶1000, Invitrogen). Samples were counterstained with 1 µ/ml DAPI. A Leukocyte Alkaline Phosphatase Kit (Sigma) was used to detect alkaline phosphatase activity.

### Microarray analysis

Total RNA was prepared with the RNeasy midi Kit (Qiagen). RNA (500 ng) was labeled with Cy3 using the Quick Amp Labeling Kit (Agilent) according to the manufacturer’s instructions. cRNA was column-purified using the RNeasy Kit (Qiagen) and hybridized to the Agilent Whole Mouse Genome Oligo Microarray (G4122A). The data were normalized and analyzed using GeneSpringGX11 (Agilent). Genes with a 4-fold change in expression between MEFs and ESCs were selected and hierarchically clustered using the centroid linkage rule.

The microarray data is MIAME compliant and the raw data have been submitted to the NCBI GEO database under the accession number GSE29441.

### *In vitro* differentiation mediated by embryoid body formation

Cell suspensions were prepared by trypsinization and 500 cells were aggregated in hanging drops of ES medium without LIF. After 3 days, embryoid bodies (EBs) were seeded onto gelatin-coated culture dishes for 4 days. Spontaneous germ-layer differentiation of EBs was assayed using markers for endoderm (AFP), mesoderm (α-SMA), and ectoderm (βIII-tubulin).

### Teratoma formation

Approximately 1 × 10^6^ cells were transplanted subcutaneously into dorsal flanks of CD-1 (ICR)-nu mice (Charles River). Tumors were isolated after 5-6 weeks and subjected to hematoxylin-eosin staining. Histological examination confirmed differentiation into all three germ layers.

### Blastocyst injection

Chimera production was performed as described previously [31]. Briefly, 4 lines of iHAC-free iPS cells (F-B1, F-C5, F-E4 and F-N7) were separately injected into blastocyst-stage embryos derived from ICR mice (CLEA Japan). Injected embryos were then transferred into pseudopregnant ICR females.

## Results

### Construction of HAC-based cell-reprogramming vectors

**Figure 1:**
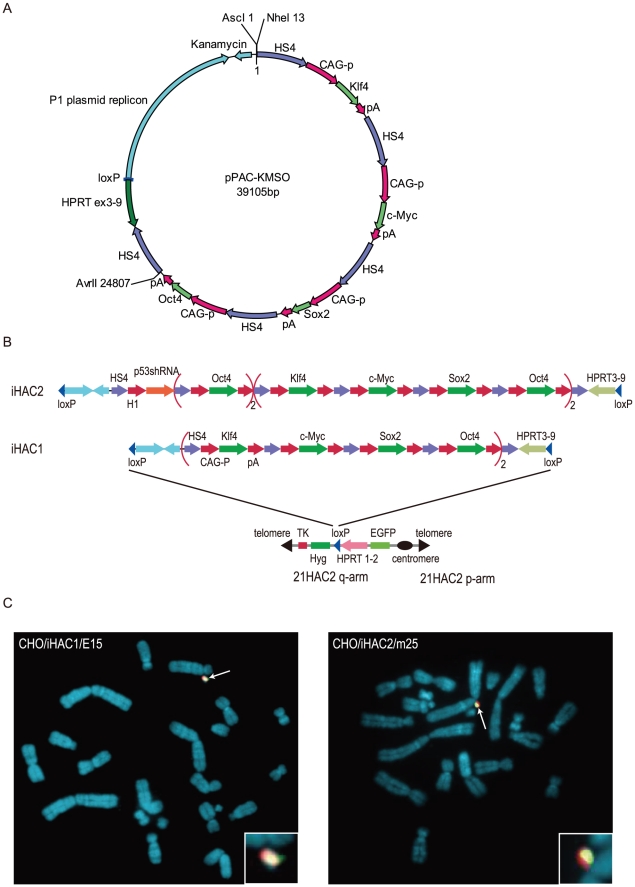
Construction of iHAC vectors. (A) A map of the reprogramming cassette constructed in a PAC vector, pPH3-9. The expression cassette comprised cDNAs for Klf4, c-Myc, Sox2 and Oct4, each under the control of a CAG promoter and flanked with HS4 insulators. The reprogramming cassette was followed by exon 3 to exon 9 of the human HPRT gene and then by a lox P site. (B) Schematic maps of the iHAC1 and iHAC2 inserts. iHAC1 has a duplicated reprogramming cassette. iHAC2 has this duplicated cassette and an additional cassette with two copies of Oct4 and a p53 shRNA construct. (C) FISH analyses of CHO/iHAC1 and CHO/iHAC2. Digoxigenin-labeled human COT-1 DNA (red) was used to detect the HAC backbone. Biotin-labeled pPAC-KMSO (green) was used to detect the reprogramming cassette in the iHACs. Chromosomal DNA was counterstained with DAPI. White arrows indicate iHAC vectors and the insets show enlarged images of the iHACs.

To introduce four reprogramming factors into recipient cells without the risk of integrating foreign genetic material into host chromosomes, we constructed HAC-based cell-reprogramming vectors. An expression cassette consisting of CAG promoter-driven cDNAs of Klf4, c-Myc, Sox2 and Oct4, each surrounded by copies of the HS4 insulator, was introduced into a modified pPAC4 vector to generate pPAC-KMSO (Fig. 1A). To achieve high expression of these four reprogramming factors, the expression cassette was duplicated, resulting in pPAC-2CAG-KMSO. To generate a more effective reprogramming cassette, two additional copies of CAG-driven Oct4 and an H1 promoter-driven p53 shRNA were inserted adjacent to the cassettes in pPAC-2CAG-KMSO, resulting in pPAC-2CAG-O2mp53sh. We chose 21HAC2 from our series of 21HAC vectors to carry these reprogramming cassettes because it encodes EGFP; therefore, recipient cells could be screened for the presence of 21HAC2 derivatives via EGFP detection. pPAC-2CAG-KMSO or pPAC-2CAG-O2mp53sh along with an expression plasmid encoding Cre recombinase was introduced into a 21HAC2-carrying Hprt-deficient Chinese Hamster Ovary (CHO) cell line, CHO(21HAC2) via co-transfection. Transformation was followed by HAT/blasticidin double selection. The HAC vectors carrying the expression cassettes from pPAC-2CAG-KMSO or pPAC-2CAG-O2mp53sh were designated iHAC1 and iHAC2, respectively (Fig. 1B). FISH, genomic PCR, and RT-PCR screens were performed to select and verify two drug-resistant CHO donor clones: CHO/iHAC1/E15 and CHO/iHAC2/mp25, which each encoded a correctly reconstituted HPRT gene, stably expressed all reprogramming factors and maintained the iHAC independently from the host chromosomes (Fig. 1C and Fig. S1). These results indicated that two iHACs (iHAC1 and iHAC2), designed to reprogram somatic cells, were structured as intended in CHO cells. These CHO cell lines can function as donor cells for microcell-mediated chromosome transfer (MMCT) of the HACs into mouse embryonic fibroblasts (MEFs).

### Derivation of iPS cells from MEFs using the iHAC vectors

**Figure 2:**
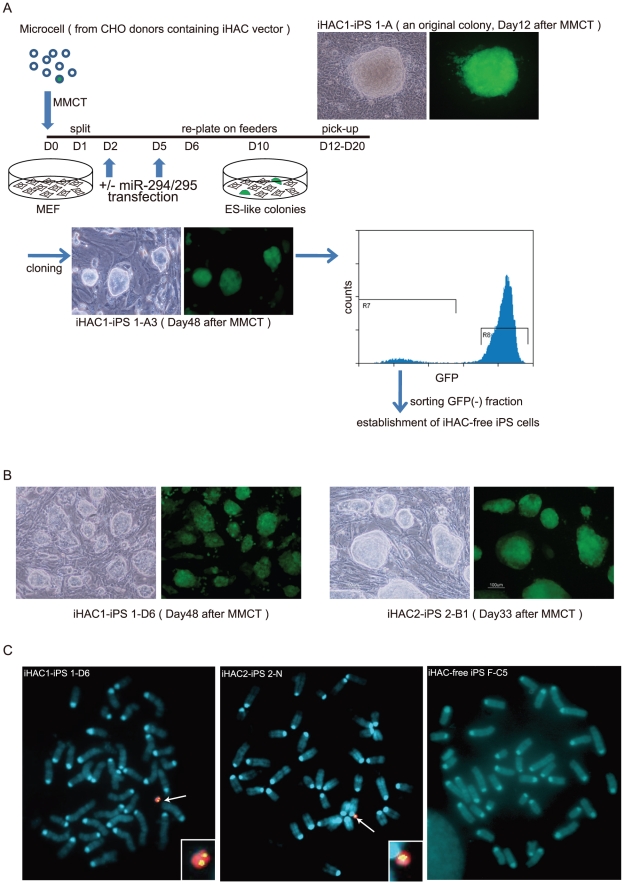
Reprogramming MEFs with iHAC1 or iHAC2. (A) Flowchart of iPS cell generation after MMCT. We screened for cells reprogrammed by iHAC vectors using two criteria: ES-like morphology and EGFP fluorescence. Packed domed colonies emerged 10 days after MMCT. After expansion, EGFP-negative fractions were sorted from isolated clones to isolate iHAC-free cells. (B) Representative bright-field and fluorescence images of iHAC1- and iHAC2-iPS clones obtained by MMCT. (C) FISH analyses of three lines, iHAC1-iPS 1-D6, iHAC2-iPS 2-N and iHAC-free iPS F-C5.

**Figure 3:**
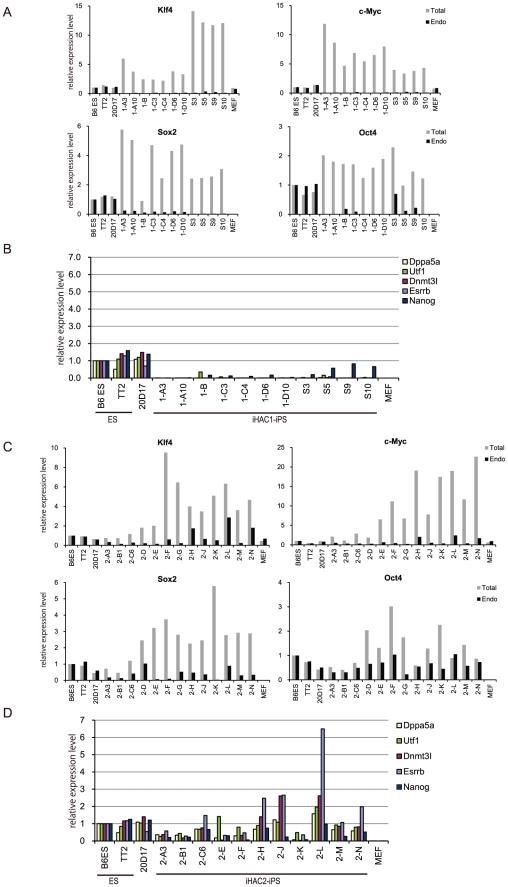
Characterization of iPS cells generated by iHAC vectors. (A, C) Quantitative RT-PCR analysis of total and endogenous reprogramming factor (Klf4, c-Myc, Sox2 and Oct4) expression in iHAC1- and iHAC2-iPS cells. Transcript levels for these genes relative to levels in B6ES cells are shown, after normalization to Nat1 expression. (B, D) Quantitative RT-PCR analyses of pluripotency markers.

To assess the reprogramming ability of iHAC1 and iHAC2, each was transferred from a CHO donor cell line into approximately 10^6^ MEFs via MMCT. A schematic of this process is shown in Figure 2A. MEFs were re-plated onto a feeder layer one day after MMCT and were treated with miR-294/295 mimics on day 2 and day 5 to raise the efficiency of reprogramming. EGFP-positive colonies with embryonic stem (ES) cell-like morphology emerged on about day 10 and were selected on days 12-20 after MMCT. The reprogramming effects of iHAC2 became apparent somewhat earlier than those of iHAC1. We obtained 7 and 21 EGFP-positive colonies from one iHAC1 and two iHAC2 transfer experiments, respectively (Table 1). Of the 7 iHAC1 colonies, 4 were expandable, and subclones of these 4 colonies were established as iHAC1-iPS cells (Fig. 2B). iHAC2-iPS subclones were also established from 13 of the 21 iHAC2 colonies. All iHAC1-iPS lines expressed Nanog and Oct4 epitopes, were alkaline phosphatase-positive (Fig. S2), and could form embryoid bodies (EBs) that differentiated into three germ layers (Fig. S3). However, based on qRT-PCR assays, expression levels of pluripotent markers were much lower in the iHAC1-iPS cells than in ES cells. Moreover, expression of transgenic reprogramming factors (Klf4, c-Myc, Sox2 and Oct4) in the iHAC1-iPS cells was higher than that of their endogenous counterparts (Fig. 3A, B). Most of the iHAC2-iPS lines expressed the pluripotent markers at levels near to those of ES cells and were more completely reprogrammed than were the iHAC1-iPS cells. Interestingly, expression of the transgenic reprogramming factors in the iHAC2-iPS cells, as in the iHAC1-iPS cells, was higher than that of the endogenous counterparts (Fig. 3C, D). FISH analyses of the iHAC-iPS lines revealed that there was one iHAC1 copy/cell in all 4 clones, and that 7 of the 13 iHAC2 clones had 1 iHAC2 copy/cell, 4 had 2 copies/cell, and 1 had more than 2 copies/cell. These 16 iHAC-iPS clones maintained the iHACs independently of the host chromosomes (Fig. 2C and Table 2). The iHAC2 vector was translocated onto a host chromosome in only one iHAC2-iPS line. These results indicated that a single copy of either iHAC induced somatic cell reprogramming, and that iHAC2 was more effective than iHAC1 in inducing this reprogramming. We also determined whether supplementation by miR-294/295 mimics were required to achieve somatic cell reprogramming by iHAC2, because the pluripotent state of iHAC2-iPS cells was highly similar to that of ES cells. Fourteen out of 22 EGFP-positive colonies (64%), obtained from the iHAC2 transfer without miR-294/295 mimics, expressed ground state pluripotency markers, Nanog and Rex1, at levels similar to those of mouse ES cells (Table 3 and Fig. S4). In the presence of miR-294/295 mimics, pluripotent clones appeared at a frequency of 57% (8 out of 14 EGFP-positive colonies), similar to that without these mimics. These results indicate that miR-294/295 mimics are not necessary for somatic cell reprogramming by the iHAC2 protocol.

**Table 1:** Summary of MMCT experiments to generate iPS cells from MEFs.

experiment No.	HAC vector	recipient	EGFP+ colony	ES-like clone	HAC-abrogated clone
Exp.1	iHAC1	10^6^ MEFs	7	4 (57%)	0 (0%)
Exp.2	iHAC2	10^6^ MEFs	6	3	2
Exp.3	iHAC2	10^6^ MEFs	15	10	5
Exp.2+3			21	13 (62%)	7 (33%)

**Table 2:** Characterization of iPS cells generated by the iHACs.

HAC vector	iPS line	subline	copy no. of iHAC	expression (qRT-PCR)		differentiation		microarray
				transgene	ES marker	*in vitro*	*in vivo*	
iHAC1	1-A	1-A3	1	high	low	3 germ layers	no tumor	Yes
		1-A10	1	high	low	3 germ layers	no tumor	N/D
	1-B		1	high	low	3 germ layers	no tumor	Yes
	1-C	1-C3	1	high	low	3 germ layers	malignant	Yes
		1-C4	1	high	low	3 germ layers	no tumor	N/D
	1-D	1-D6	1	high	low	3 germ layers	malignant	Yes
		1-D10	1	high	low	3 germ layers	malignant	N/D
iHAC2	2-A	2-A3	1	high	moderate	N/D	N/D	N/D
	2-B	2-B1	1	high	moderate	N/D	N/D	Yes
	2-C	2-C6	1	high	moderate	N/D	N/D	Yes
	2-D		2	high	moderate	N/D	N/D	N/D
	2-E		1	high	moderate	N/D	N/D	Yes
	2-F		2	high	moderate	N/D	N/D	N/D
	2-G		translocated	high	moderate	N/D	N/D	N/D
	2-H		2	high	moderate	N/D	N/D	N/D
	2-J		1	high	moderate	N/D	N/D	N/D
	2-K		2	high	moderate	N/D	N/D	N/D
	2-L		1	high	moderate	N/D	N/D	N/D
	2-M		2	high	moderate	N/D	N/D	N/D
	2-N		1	high	moderate	N/D	N/D	Yes

N/D not determined.

**Table 3:** Efficiency of iPS cell generation from MEFs using iHAC2 with or without miR-294/295.

experiment No.	HAC vector	recipient	miR-294/295	EGFP+ colony	clone ID	pluripotent clone
Exp.4	iHAC2	10^6^ MEFs	-	15	A1 - A15	7
Exp.5	iHAC2	10^6^ MEFs	-	7	B1 - B7	7
Exp.4+5				22		14 (64%)
Exp.6	iHAC2	10^6^ MEFs	+	14	C1 - C14	8 (57%)

### Establishment of integration-free iPS cells

**Figure 4:**
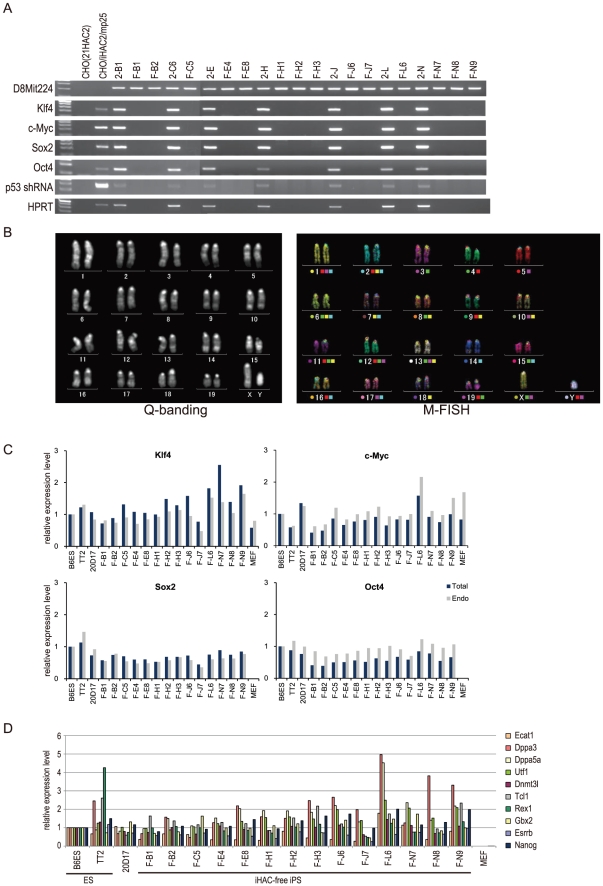
Establishment of iHAC-free iPS cells. (A) Genomic PCR of individual transgenes in cells from an EGFP-negative fraction of iHAC2-iPS cells was compared with that of the parental clones. D8Mit224 was used as an internal control. (B) Cytogenetic analysis of iHAC-free iPS cells. Representative karyotypes of iHAC-free iPS F-C5 (Q-banding) and F-N7 (M-FISH) show normal karyotypes after loss of iHAC2. (C) Quantitative RT-PCR of total and endogenous reprogramming factor expression. (D) Quantitative RT-PCR of pluripotency markers. Transcript levels of 10 pluripotent genes were normalized to Nat1; transcript levels in iHAC-free iPS cells were compared with those in B6ES cells.

Sustained transgene expression can affect the function of iPS cells and can lead to deleterious effects, such as tumorigenesis [32], [33]. Therefore, we established iHAC-free iPS clones from the iHAC-iPS cells by isolating EGFP-negative clones. During host cell division under non-selecting conditions, the HAC vectors are lost spontaneously at a low frequency (1×10^-5^). An EGFP-negative fraction from one iHAC1-iPS line (clone 1-D6) (up to 10^5^ cells) was harvested by FACS (MoFlo XDP, Beckman Coulter), plated onto a feeder layer, and re-cloned based on morphological criteria. EGFP-negative colonies (n = 4) were selected randomly and assayed for the presence or absence of the iHAC1 vector. FISH and genomic PCR analyses revealed that all 4 EGFP-negative clones (clones S3, S5, S9 and S10) retained the iHAC1 vector (Table 4), and qRT-PCR analysis indicated that all transgenes were expressed (Fig. 3A). These results indicated that the iHAC1-iPS cells were only partially reprogrammed and that their pluripotent status was dependent on continued transgene expression from the iHAC1. Thus, iHAC1-free iPS lines were not established from the iPS cells, and the EGFP-negative clones probably resulted from epigenetic silencing of the EGFP gene. In contrast, iHAC2-free iPS cells were isolated from 7 of the original12 iHAC2-iPS clones by selecting for EGFP-negative cells. The iHAC2 vector was not detected by FISH or genomic PCR in 14 sub-clones (Fig. 2C and Fig. 4A); therefore, we concluded that the iHAC2 vector was absent from the EGFP-negative fractions of the iPS cell lines. Cytogenetic analyses with Q-banding and M-FISH confirmed that the iHAC2-free clones had a normal karyotype (Fig. 4B). Furthermore, expression levels of the endogenous reprogramming factors (Klf4, c-Myc, Sox2 and Oct4) were similar in iHAC2-free clones and mouse ES cells (Fig. 4C). These results are summarized in Table 4.

**Table 4:** Characterization of iPS cells isolated from the GFP-negative population by FACS.

parental clone	sorted clone	presence of HAC	expression (qRT-PCR)		differentiation		chimera	microarray
			transgene	ES marker	*in vitro*	*in vivo*		
1-D6	S3	present	high	low	3 germ layers	teratoma	N/D	N/D
	S5	present	high	low	3 germ layers	teratoma	N/D	N/D
	S9	present	high	low	3 germ layers	teratoma	N/D	N/D
	S10	present	high	low	3 germ layers	teratoma	N/D	N/D
2-B1	F-B1	absent	none	high	3 germ layers	teratoma	chimera	Yes
	F-B2	absent	none	high	3 germ layers	teratoma	N/D	N/D
2-C6	F-C5	absent	none	high	3 germ layers	teratoma	chimera	Yes
2-E	F-E4	absent	none	high	3 germ layers	teratoma	chimera	Yes
	F-E8	absent	none	high	3 germ layers	N/D	N/D	N/D
2-H	F-H1	absent	none	high	3 germ layers	N/D	N/D	N/D
	F-H2	absent	none	high	3 germ layers	teratoma	N/D	N/D
	F-H3	absent	none	high	3 germ layers	N/D	N/D	N/D
2-J	F-J6	absent	none	high	3 germ layers	N/D	N/D	N/D
	F-J7	absent	none	high	3 germ layers	teratoma	N/D	N/D
2-L	F-L6	absent	none	high	3 germ layers	N/D	N/D	N/D
2-N	F-N7	absent	none	high	3 germ layers	teratoma	chimera	Yes
	F-N8	absent	none	high	3 germ layers	N/D	N/D	N/D
	F-N9	absent	none	high	3 germ layers	N/D	N/D	N/D

N/D not determined.

### Pluripotency of iHAC-free iPS cells

**Figure 5:**
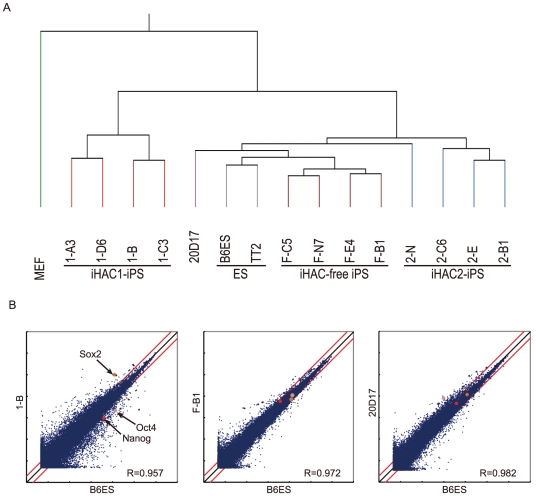
Global gene expression analysis of iHAC1-, iHAC2-, and iHAC-free iPS cells. (A) Unsupervised hierarchical clustering of global gene expression profiles from ES cells, iPS cells generated by retroviral or iHAC vectors, and MEFs. (B) Scatter plots of microarray data comparing ES cell line B6ES to iHAC1-iPS 1-B, iHAC-free iPS F-B1 and retroviral iPS 20D17 cells. Highlighted marks indicate core pluripotent markers, Nanog, Oct4 and Sox2. Red lines indicate two-fold changes in expression between samples.

**Figure 6:**
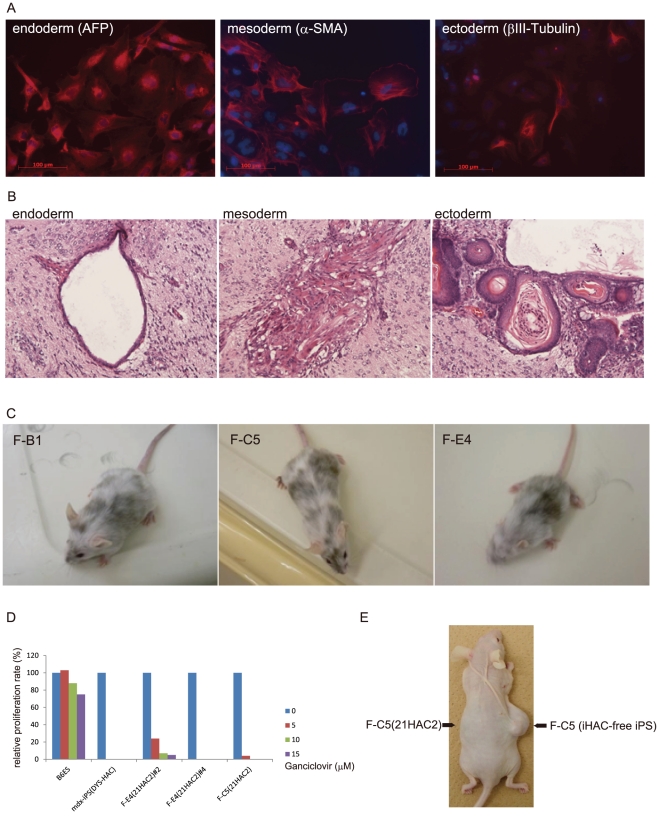
Pluripotency of iHAC-free iPS cells and a HAC safeguard system. (A) *in vitro* differentiation mediated by EB formation. (B) Representative images of various tissues present in teratomas derived from iHAC-free iPS F-C5 cells. (C) Live-born chimeras from iHAC-free iPS clones. (D, E) Confirmation of the HAC safeguard system. Growth of iHAC-free iPS clones re-transformed with 21HAC2 or with a retroviral iPS clone harboring DYS-HAC was inhibited by GCV *in vitro* (D). Parental iHAC-free iPS F-C5 and F-C5/21HAC2 were subcutaneously transplanted into nude mice. 30 mg/kg GCV was then administered intraperitoneally for 24 consecutive days (E).

To assess the pluripotent state of the iHAC-free iPS cells, we performed qRT-PCR analyses using a variety of stem cell markers. The iHAC-free iPS cells expressed 12 pluripotency markers, including Sox2 and Oct4, at levels similar to those of mouse ES cells (Fig. 4C, D). Furthermore, to assess whether absence of transgenes enhanced the pluripotent state of the iHAC-iPS cells, we compared the global gene expression profiles of mouse ES cells, the iHAC1-iPS cells, the iHAC2-iPS cells, and the iHAC-free iPS cells. Unsupervised hierarchical clustering and scatter plots showed that the iHAC-free iPS cells were more similar to ES cells than to the iHAC1-iPS cells and the iHAC2-iPS cells, and that the iHAC1-iPS cells were most distant from ES cells (Fig. 5A, B). All 7 iHAC-free iPS clones examined (F-B1, F-B2, F-C5, F-E4, F-H2, F-J7 and F-N7) also had differentiation potential in both *in vitro* (EB-based) and *in vivo* (teratoma) assays. Furthermore, 4 of 4 iHAC-free iPS clones tested gave rise to live chimeras (Fig. 6A-C). Although it remains to be determined whether germ line transmission in adult chimeras occurs, it can be said that the iHAC-free iPS cells were pluripotent.

### A safeguard system

Our 21HAC vectors carry a suicide gene, *HSV*-TK, to eliminate cells with undesirable or unexpected phenotypes (e.g., tumor development) [18]. To verify that this safeguard system functions in iPS cells, we introduced a 21HAC2 vector into Duchenne muscular dystrophy (DYS) model (mdx)-iPS cells previously generated from MEFs of mdx mice by the retroviral transduction of four reprogramming factors [34]. The mdx-iPS(DYS-HAC) cells were produced by the transfection of 21HAC2, harboring *HSV*-TK, and a full-length Dystrophin locus. GCV, administered consecutively over 32 days, selectively repressed advancement of teratomas from the mdx-iPS(DYS-HAC) cells, but not of control mdx-iPS cells, and administration of PBS had no effect on either cell line (Fig. S5). Next, we assessed the effects of GCV on iHAC-free iPS cells (derived from the iHAC2-iPS cells) that were transferred with 21HAC2. The 21HAC2 was introduced into iHAC-free iPS clones F-C5 and F-E4. Isolated iPS clones carrying 21HAC2 were tested for sensitivity to GCV. The iPS clones exhibited *in vitro* growth retardation in 5 µM GCV, which is the effective dosage for mouse ES cells carrying 21HAC2 [18]. Advancement of a teratoma from F-C5/21HAC2, but not from F-C5, was inhibited by consecutive administration of GCV (Fig. 6D, E). Therefore, the *HSV*-TK suicide system of the HAC vectors operated in iPS cells that were generated by either a retrovirus vector or the iHAC2 vector. In addition, the iPS cells carrying 21HAC2 differentiated into all three germ layers in the teratoma and contributed to chimera development (data not shown), suggesting that the re-introduced 21HAC2 vector did not affect the pluripotency of iPS cells.

## Discussion

We demonstrate that a HAC vector containing expression cassettes for four reprogramming factors and a p53-knockdown construct efficiently reprogrammed somatic cells to pluripotency. In addition, we established integration-free iPS cells derived from these reprogrammed cells. Inserting all expression constructs into a defined cloning site on the HAC vector, which was maintained stably and independently of host chromosomes in cells, resulted in homogenous expression levels of the transgenes in HAC donor and recipient cells. Once configuration of an expression cassette in the HAC vector is optimized for the generation of iPS cells, the resulting uniformity of transgene expression in target cells is an advantage in promoting reprogramming efficiency and reducing clonal variation in the resulting iPS cells. Thus, the HAC-based reprogramming strategies are expected to be more effective in establishing homogenous iPS clones than other methods, including DNA transfection and viral transduction, which are both unable to regulate the quantity of xeno-products in modified cells. Nonetheless, the transfer rate of HAC vectors via MMCT is relatively low, i.e., 10^-5^. To overcome this drawback, our protocol was enhanced with two procedures. First, to sustain high expression levels of individual reprogramming factors, each factor was surrounded with insulators. Second, to potentiate the reprogrammed state, miR294 cluster mimics, which promote induced pluripotency [35], were added after MMCT. Indeed, although the overall efficiency of reprogramming by our iHAC strategy was approximately 0.001%, more than half of iHAC-bearing cells developed an ES-like phenotype (iHAC1, 57%; and iHAC2, 62%). Furthermore, vector-free, transgene-free iPS cells were established from a third of the iHAC2-iPS lines. Notably, the effect of iHAC2 on the generation of iPS cells was no longer dependent on the addition of miR294 cluster mimics, because the pluripotent state induced by iHAC2 alone was sufficiently high. Therefore, the HAC vector system did facilitate somatic cell reprogramming by homogenous expression of the transgenic reprogramming factors and established vector-free, transgene-free iPS cells, which are suitable for clinical applications. Nevertheless, the MMCT frequency needs to improve. An improved MMCT technology may enhance the frequency of reprogramming by iHAC (50-100 times) [36].

All iHAC1-iPS cells satisfied some criteria of pluripotency (e.g., alkaline phosphatase staining, EB formation, ability of EB cells to differentiate into three germ layers); however, the iHAC1-iPS cells expressed only low levels of various pluripotent markers, indicating only partial reprogramming. In contrast, expression of pluripotent markers in most of the iHAC2-iPS cells was significantly upregulated and close to that in ES cells. These results were consistent with previous studies, which demonstrated that increasing Oct4 expression relative to the other three reprogramming factors [37] and suppression of the p53 pathway [25]-[29] resulted in enhanced reprogramming efficiency. Notably, in both iHAC1-iPS and iHAC2-iPS cells, gene silencing of the transgenic reprogramming factors was incomplete or nonexistent because each reprogramming factor was surrounded by multiple copies of the insulator. Moreover, expression levels of anti-proliferative genes in the iHAC1-iPS and the iHAC2-iPS cells were similar to those in ES cells, and were not upregulated (Fig. S6). Therefore, the major cause of the partial reprogramming in the iHAC1-iPS cells, but not the iHAC2-iPS cells, may be inadequate activation of endogenous pluripotent genes rather than sustained expression of transgenes or induction of anti-proliferative genes. Recently, it has been demonstrated that Nanog drives partially reprogrammed cells into ground state pluripotency [38]. Therefore, we can assume that p53 shRNA and the additional copies of Oct4 encoded by iHAC2 may have contributed to consolidating connections between core transcription factor networks and the enhanced expression of genes like Nanog [39]. This hypothesis is supported by the evidence that supplementation of miR294/295 was no longer required for the iHAC2 reprogramming protocol. These results indicate that transgene integration into a defined cloning site on a HAC vector may also be useful for screening other reprogramming factors that improve the quality of iPS cells and increase overall efficiency.

**Figure 7:**
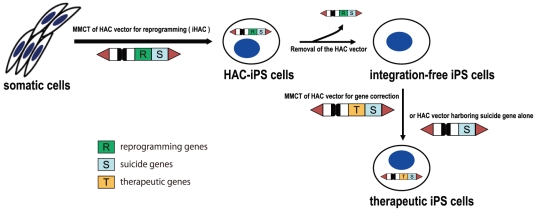
Regenerative medicine strategy using HAC vectors. Our proposed strategy to generate patient-specific iPS cells with therapeutic transgenes carried by HAC vectors is depicted. Integration-free iPS cells are created from patient-specific somatic cells by HAC-mediated reprogramming (iHAC). Subsequently, correction of a gene lesion is achieved by the addition of a HAC with a therapeutic gene and a suicide gene.

Removal of potential obstacles, such as persistent exogenous genes or chemicals, the maintenance of normal cellular functions and the preservation of genome integrity are fundamental to the application of iPS cells in regenerative medicine. Here, we demonstrated that a HAC vector can mediate somatic cell reprogramming and that transgene-free, vector-free iPS cells can be obtained from iHAC2-iPS cells using simple FACS sorting; in contrast, other systems require prolonged culture, vector excision and drug selection to obtain integration-free iPS cells. Furthermore, the iHAC-free iPS cells generated using iHAC2 did not exhibit chromosomal aberrations, although the present version of iHAC2 contained a p53-knockdown construct. Thus, the strategy of using HAC vectors to generate integration-free iPS cells might be valuable. Moreover, the HAC vector itself was safe because it was maintained independently of host chromosomes, and importantly, chimeric mice were produced from mouse ES/iPS cells harboring a HAC vector [18], [19], [34]. We have demonstrated that HAC vectors are able to carry therapeutic genes, especially genomic loci larger than 1 Mb, and that they are able to correct multiple cellular defects in target cells without transgene integration [20], [34]. As illustrated in Figure 7, our overall strategy for regenerative medicine using HAC vectors equipped with one or more suicide genes as a safeguard system may facilitate the generation of patient-specific iPS cells that complement genetic traits causing diseases without risking genomic alteration and other undesirable outcomes.

## Supporting Information

Figure S1 **Transgene expression in CHO donor cells.** Expression of the four reprogramming factors contained in the iHAC vectors was confirmed by RT-PCR. In qRT-PCR assays, transcript levels of transgenes were standardized to Gapdh. Transcript levels in CHO/iHAC2/mp25 were compared to levels in CHO/iHAC1/E15.
(EPS)

Figure S2 **Characterization of iHAC1-iPS cells.** (A) Alkaline phosphatase staining of iHAC1-iPS 1-A10, 1-C3, 1-D10 and mouse ES TT2 cells. (B, C) Immunostaining of pluripotent markers. iPS cells with the iHAC1 vector and that were positive for EGFP (green) were stained for Nanog (B) and Oct4 (C).
(EPS)

Figure S3 ***In vitro*** **differentiation of iHAC1-iPS cells.** (A) EB formation. (B) Phase-contrast images showing differentiated cells derived from EBs. (C) Immunostaining confirming that iHAC1-iPS cells differentiated into each germ layer *in vitro*.
(EPS)

Figure S4 **Expression levels of pluripotent markers, Nanog and Rex1.** Pluripotency of iHAC2-iPS cells generated without miR-294/295 was compared to that of iHAC2-iPS cells with miR-294/295 by quantitative RT-PCR analyses of Nanog and Rex1 expression. Transcript levels were standardized to Nat1 and normalized to B6ES cells.
(EPS)

Figure S5 **Anti-proliferative effect of GCV on** ***HSV*** **-TK-containing mdx-iPS(DYS-HAC) cells** ***in vivo*** **.** Parental mdx-iPS cells and mdx-iPS(DYS-HAC) cells were subcutaneously transplanted into nude mice, then GCV was administered intraperitoneally. Tumor volume was calculated using the formula [width (mm)]×[length (mm)]×[depth (mm)]. Administration of PBS was used as a control.
(EPS)

Figure S6 **Expression levels of p53, Ink4a, Arf and Cip1.** Quantitative RT-PCR analyses of p53, Ink4a, Arf and Cip1 levels in iHAC1- and iHAC2-iPS cells were compared with levels in parental MEFs and mouse ES cells. Transcript levels were standardized to Nat1 and normalized to B6ES cells.
(EPS)

Table S1 **Primers used for PCR analyses.**
(XLS)
